# On the Physiological Action of Thaline

**Published:** 1890-01

**Authors:** A. Dagenais

**Affiliations:** Buffalo, N. Y.


### ON THE PHYSIOLOGICAL ACTION OF THALINE.

Translated from *L'Abeille Médicale*.

By A. DAGENAIS, M. D., Buffalo, N. Y.

M. Robin presented to the Academy the résumé of his researches on the physiological action of thaline, showing that its salts have the same actions on the blood as the salt of kairine. Thaline destroys the hemoglobine; it is a poison to the red globules; it diminishes the respiratory capacity of the blood. If a few drops of sulphate of thaline solution are mixed with blood, the red color disappears, the blood takes a brown chocolate color, similar to the changes observed by its mixture with kairine. There is also a destruction, nearly complete, of hemoglobine; the two characteristic raies of hemoglobine disappear, to make room for those of methemoglobine. The fall of temperature which clinicians—Jaksch, of Vienna, in particular,—have obtained in certain pyrexia, following the use of thaline salts, was the result of the destruction of the hemoglobine of the blood. The modifications that M. Robin has remarked in the organic exchanges following the thalinic medication are attributed to the same cause, to the disposition of the greatest part of vehicles intended to carry oxygen to the tissues.

M. Browardel said that he was one of the first to warn the profession against the use of the salts of thaline. This communication from M. Robin confirms what he (Browardel) said before.

